# The Intensity of Internal and External Attention Assessed with Pupillometry

**DOI:** 10.5334/joc.336

**Published:** 2024-01-09

**Authors:** Damian Koevoet, Marnix Naber, Christoph Strauch, Stefan Van der Stigchel

**Affiliations:** 1 Experimental Psychology, Helmholtz Institute, Utrecht University, The Netherlands

**Keywords:** Visual working memory, Pupillometry, Internal attention, External attention, Intensity

## Abstract

Not only is visual attention shifted to objects in the external world, attention can also be directed to objects in memory. We have recently shown that pupil size indexes how strongly items are attended externally, which was reflected in more precise encoding into visual working memory. Using a retro-cuing paradigm, we here replicated this finding by showing that stronger pupil constrictions during encoding were reflective of the depth of encoding. Importantly, we extend this previous work by showing that pupil size also revealed the intensity of internal attention toward content stored in visual working memory. Specifically, pupil dilation during the prioritization of one among multiple internally stored representations predicted the precision of the prioritized item. Furthermore, the dynamics of the pupillary responses revealed that the intensity of internal and external attention independently determined the precision of internalized visual representations. Our results show that both internal and external attention are not all-or-none processes, but should rather be thought of as continuous resources that can be deployed at varying intensities. The employed pupillometric approach allows to unravel the intricate interplay between internal and external attention and their effects on visual working memory.

## 1. Introduction

Attention allows for selectively processing the most relevant information within the rich visual world. Besides the selection of relevant information from the external world (e.g. looking for an apple in the grocery store), attention can also select information from temporarily internally stored representations in visual working memory (VWM; e.g. remembering you need to buy a banana as well). As such, attention shifts to selectively enhance items on a perceptual (i.e. external attention) or memory level (i.e. internal attention) to guide behavior (Chun et al., 2011; Gresch et al., 2023; Griffin & Nobre, 2003; Hautekiet et al., 2023; Heuer et al., 2020; Landman et al., 2003; Oberauer, 2019; Olivers & Roelfsema, 2020; Souza & Oberauer, 2016; van Ede, 2020; van Ede & Nobre, 2023; Verschooren & Egner, 2023).

One aspect of attention that is relatively unexplored concerns how *intensely* it is deployed (Miller et al., 2019; Unsworth & Miller, 2021). A possible reason for this lacuna in the literature is that traditional behavioral outcomes do not allow to determine the intensity of attention on a trial-by-trial basis, as such outcomes necessitate the calculation of summary values across conditions (e.g. accuracy in % correct or mean response times). The limitation of these behavioral measures is further exacerbated if internal attention is assessed, since it can be difficult to explicitly disentangle its effects from external attention (Koevoet, Strauch, Van der Stigchel, et al., 2023). Importantly, the ultimate quality of VWM representations is shaped by the integrative outcome of the intensity of external and internal attention. VWM encoding is strongly modulated by attention (e.g. Awh et al., 2006; Blom et al., 2016; Koevoet, Naber, et al., 2023) and dual-tasks have been shown to disrupt VWM prioritization (e.g. Janczyk & Berryhill, 2014; Lin et al., 2021). Thus, encoding and prioritization consume attentional resources and having more of such resources available for the task leads to improved VWM precision.

Pupil size holds promise as a physiological measure to effectively capture and delineate between the intensities of external and internal attention. Here, internal attention exclusively refers to the prioritization of material stored in VWM (Chun et al., 2011; van Ede & Nobre, 2023). We have recently proposed that different pupillary responses capture distinct operations of VWM (Koevoet, Strauch, Van der Stigchel, et al., 2023; Strauch, Wang, et al., 2022). For instance, the pupil orienting response and a later pupil dilatory component index encoding (external attention) and prioritization (internal attention), respectively. The pupil orienting response occurs within 200–700 ms following the onset of a visual stimulus and manifests as a brief dilation followed by a prominent constriction (Koevoet, Strauch, Van der Stigchel, et al., 2023; Lynn, 2013; Nieuwenhuis et al., 2011; Strauch, Romein, et al., 2022; Strauch, Wang, et al., 2022; Wang & Munoz, 2015). Specifically the pupil orienting constriction is linked to the depth of sensory processing, and thus the intensity of external attention (Barbur & Thomson, 1987; Binda & Murray, 2015; Naber et al., 2013; Strauch, Wang, et al., 2022). Building on this notion, our previous findings demonstrate that stronger orienting constrictions predict the amount and the precision of information committed to VWM (Koevoet, Naber, et al., 2023).

Pupillometric studies have leveraged the pupil light response (PLR) to study *what* is externally or even internally attended (Hustá et al., 2019; Unsworth & Robison, 2017; Zokaei et al., 2019). The PLR entails pupil dilations and constrictions in response to external dark and bright visual stimuli, respectively. Through the PLR, pupil size reveals what is externally attended: Attending dark items leads to larger pupils than when attending bright items (Binda & Gamlin, 2017; Binda et al., 2014; Mathôt & Van der Stigchel, 2015; Mathôt et al., 2013; Strauch, Romein, et al., 2022). Remarkably, Zokaei et al. (2019) recently showed that pupil size also reveals the focus of internal attention. In this study participants encoded bright and dark items during a VWM task. Through an auditory retro-cue, participants were instructed to internally attend to a specific brightness stored in memory. Crucially, whenever participants shifted internal attention toward a dark item, the pupil dilated more than whenever a bright item was prioritized internally. The magnitude of this PLR-modulated prioritization effect predicted participants’ working memory precision, directly linking it to behavior. Additionally, Unsworth and Robison (2018) found that internally prioritizing information in working memory elicited a pupil dilation, even irrespective of brightness (Unsworth & Robison, 2018). This response likely reflects (mental) effort akin to dilations during (V)WM maintenance (Beatty, 1982; Kahneman, 1973; Koevoet, Strauch, Van der Stigchel, et al., 2023; Strauch, Wang, et al., 2022), but this effect was specific to the internal prioritization process. The observed dilation during prioritization therefore reveals that shifting internal attention requires effort, making pupillometry a potentially valuable outcome measure for quantifying the intensity of such shifts. Note that we here consider the intensity of internal attention to be the sum of sub-processes such as internal orienting, selection and enhancement of stored representations (see van Ede & Nobre, 2023) because pupil dilation cannot delineate between these sub-processes. However, the precise relationship between behavioral outcomes and the brightness-independent dilation response when deploying internal attention remains unexplored.

Here, we reanalyzed data from a retro-cuing VWM task (Wilschut & Mathôt, 2022) to address three main questions. First, we aimed to replicate our previous finding which showed that more pronounced pupil constrictions during encoding predict more precise VWM representations (Koevoet, Naber, et al., 2023). Second, we asked whether pupil size captures the intensity of internal attention. Building on the link between the PLR-based internal prioritization effect and behavior (Unsworth & Robison, 2018; Zokaei et al., 2019), we hypothesize that this general pupil dilation response reflects the intensity of internal attention and, consequently, predicts the precision of VWM representations (Koevoet, Strauch, Van der Stigchel, et al., 2023). Third, if the pupil response components indeed reflect distinct VWM operations, both the constriction during encoding and the dilation during prioritization should predict the precision of VWM representations. We predict that both of these components shape the quality of stored material on a trial-by-trial basis.

## 2. Methods

### 2.1 Procedure

**Figure 1:**
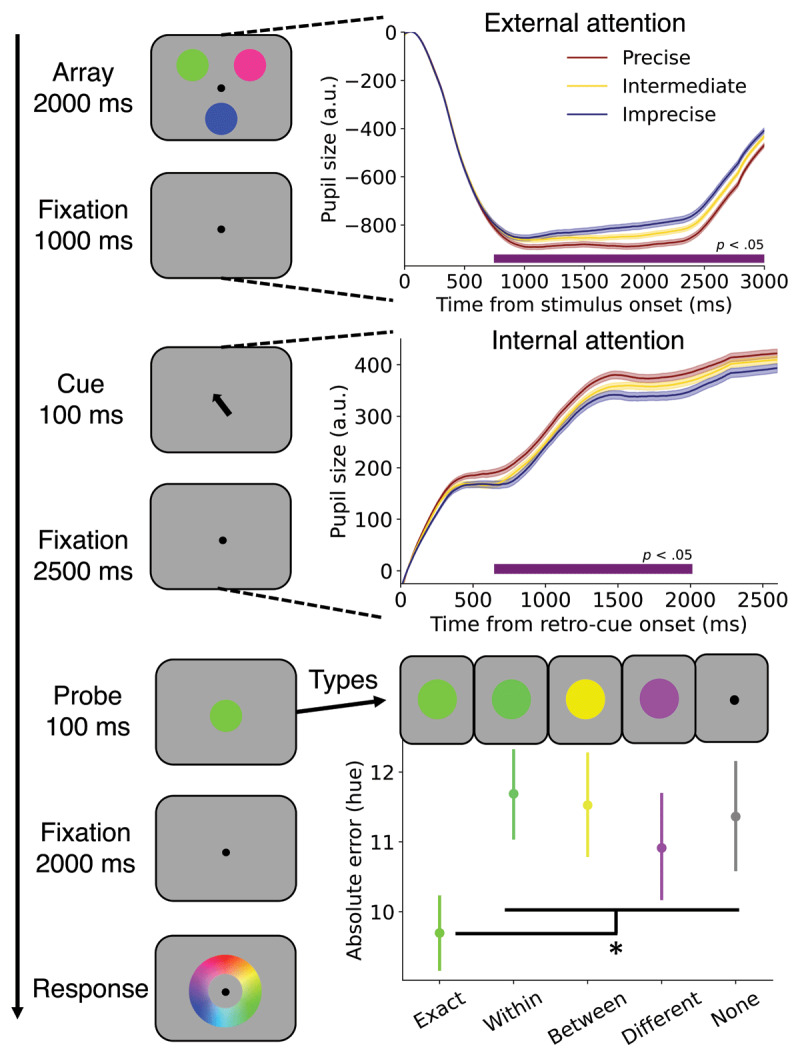
Schematic overview of the procedure and the main results. Left: Procedure from top to bottom, see Methods for details. Top right: Pupil constriction was more pronounced during more precise encoding into VWM as indicated by smaller absolute hue errors. For visualization only, three groups were created for the pupil trace plots but analyses were conducted using absolute error as a continuous variable. Horizontal purple lines indicate significant effects of absolute error on pupil size (*p* < .05). The X-axis represents time (ms) and the Y-axis holds pupil size (arbitrary units). Middle right: Stronger dilation during prioritization leads to more precise VWM representations (same conventions as above). Bottom right: Probe conditions differed in absolute hue errors. All error shadings and bars reflect standard errors of the mean. *p < .05.

Thirty-one participants took part in the experiment reported in Wilschut and Mathôt (2022) (Figure 1; see the original paper for the full details). The original experimental procedure was approved by the ethics committee of the department of psychology at the University of Groningen (study approval code: PSY-2021-S-0321).

Briefly, participants encoded three different colored circles (2000 ms; 1.7° radius), maintained these colors for 1000 ms, and the spatial location of one color was then retrocued (100% valid) for reproduction (100 ms). After another delay period (2500 ms), one of five different task-irrelevant probes was presented for 100 ms: Exactly the same as the retro-cued colors (e.g. green), a color from the same category (e.g. lighter green), a different color (e.g. yellow), a color on the opposite side of the color wheel (e.g. purple) or no probe. These probes were used to investigate how different types of colors would affect pupil responses and bias eventual VWM reports in the original paper (Wilschut & Mathôt, 2022). Lastly, after a final delay period (2000 ms), participants reported the color of the retro-cued stimulus on a color wheel with a mouse click which ended the trial. Participants were instructed to respond as precisely as possible, and were awarded points based on their precision (for details see Wilschut & Mathôt, 2022).

The experiment consisted of a total of 190 trials equally distributed across probe conditions (38 per condition) in a mixed order, which were preceded by five practice trials. Throughout the experiment, gaze position and pupil size were measured monocularly using an EyeLink 1000 desktop mount (SR Research, Mississauga, ON, Canada) at 1000Hz.

### 2.2 Data Processing and Analyses

Whereas Wilschut and Mathôt (2022) focused on the pupillary constriction response to the probe (see original paper for details), we were interested in the intensity of external and internal attention. Therefore, we only analyzed pupil size data during the encoding (i.e. color stimuli) and prioritization periods in the task (i.e. retro-cue).

Data processing and analyses were performed using custom Python scripts (version 3.9.7). Analyses were based on previous work (Koevoet, Naber, et al., 2023; Strauch, Wang, et al., 2022). Practice trials were discarded from all analyses. Blinks in the pupil data were interpolated (Mathôt & Vilotijević, 2022), downsampled to 100Hz and remaining missing data were linearly interpolated. To isolate the effects of external and internal attention, we picked a time window of 0–3000 ms after stimulus onset and a separate later window of 0–2600 ms upon retro-cue onset. Subtractive baseline correction was applied on both windows using the median pupil size of the first 100 ms of each respective window (Mathôt et al., 2018) – this interval was also used to compute baseline pupil size (see Discussion). The absolute difference in hue between the memorized and reported color was used as an index of VWM precision. We chose this index of VWM precision to be consistent with previous work (e.g. Gresch et al., 2023; Koevoet, Naber, et al., 2023). Unlike Wilschut and Mathôt (2022), we did not consider categorical differences between colors (i.e. red vs. blue) since this is a more rigid and less sensitive outcome measure of VWM precision. To assess whether and when pupil size differs during encoding and/or prioritization, linear mixed-effects (LME) models were used to analyze pupil size over time for the internal and external windows (every 10 ms; R formula: pupil size ∼ absolute hue error + probe type + (1+probe type|participant). Probe type was added as a covariate and we modelled random slopes for the probe conditions for these analyses over time (Barr, 2013) to ensure this did not drive potential effects of interest. Note that for visualization only (see Figure 1), trials (5890 in total) were split into three precision groups: precise (≤5° error), intermediate (>5 and ≤15° error) and imprecise (>15° error).

To determine whether these pupillary dynamics predict the precision of VWM representations on a trial-by-trial basis, another LME was conducted. For every trial, the absolute error of the response (in hue) was calculated as a measure of the precision of VWM content. Next, we computed average pupil size within 1000–2000 ms after stimulus onset and within 1000–2000 ms after retro-cue onset, respectively. These windows were determined based on the results from the analysis of pupil size over time to ensure external and internal attention were captured accurately. These pupillary dynamics were then used to predict the precision of VWM representations on a trial-by-trial basis. Due to problems with convergence during Akaike information criterion based selection (AIC), we chose not to include random slopes during model selection (R formula for selected model: absolute hue error ∼ encoding constriction + prioritization dilation + trial number + probe type + (1|participant); see Supplementary Materials for full details). For all analyses, effects were deemed significant whenever *t* > 1.96 corresponding to *α* = 0.05.

## 3. Results

### 3.1 Pupil Constriction Reveals Encoding Precision

We first investigated whether pupil size would capture the intensity of external attention during encoding of the three colors. Based on our previous findings, we hypothesized that stronger pupil constrictions would accompany more precise reports. Indeed, pupil size was significantly smaller already 750 ms after stimulus onset when more precise responses were given at the end of the trial (top right in Figure 1), and this effect lasted throughout the rest of the time window. This replicates our previous findings (Koevoet, Naber, et al., 2023), although the response profile of pupil size differed considerably from those previously reported. The here observed pattern includes a relatively late re-dilation after constriction that is possibly caused by the relatively long presentation of to-be-encoded stimuli (2000 ms vs. 500 ms in Koevoet, Naber, et al. (2023)), leading to sustained rather than transient pupil constrictions. This is likely driven by the average higher brightness of the color stimuli compared with the background, leading to a sustained PLR until the offset of the stimuli at 2000 ms, which in turn elicits a re-dilation. Although the shape of the trace differs compared to previous work, we nevertheless replicate the core finding that smaller pupils during encoding reflect more precise uptake into VWM.

### 3.2 Intensity of Internal Attention Is Reflected in Pupil Dilation

Next, we assessed whether pupil size also indexes the intensity of internal attention (middle right in Figure 1). Previous work showed that internal prioritization of memorized material leads to a pupillary dilation (Robison et al., 2023; Unsworth & Robison, 2018). We hypothesized that the extent of this dilation betrays how strongly internal attention is deployed. In line with our hypothesis, the pupil dilated more upon prioritization whenever VWM representations were preciser. More specifically, this pupillary dilation effect established itself around 650 ms and was sustained until 2010 ms after retro-cue onset. This shows that internal attention is deployed at different intensities, enhancing VWM precision at varying degrees.

### 3.3 Internal and External Attention Jointly Predict Precision

**Figure 2:**
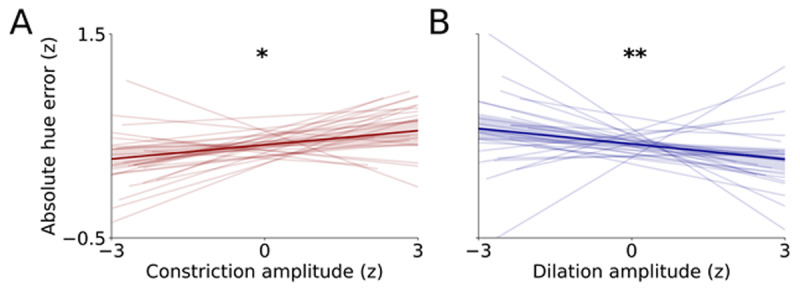
Relationships between visual working memory precision and **A)** encoding constriction amplitude (external attention), and **B)** prioritization dilation amplitude (internal attention). Thin lines represent linear fits per individual and the thick like represents the relationship across all data. Shaded error bars represent bootstrapped 95% confidence intervals. Y-axes are shared between A and B. To account for individual differences, data were normalized to robust z-scores by subtracting the median and dividing by the median absolute deviation for each datapoint per participant (Rousseeuw & Hubert, 2011). *p < .05, **p < .01.

The analyses above established that the intensity of both external and internal attention are captured by pupillary responses, but how do these forms of attention shape the quality of VWM representations? To address this directly, we analyzed whether pupillary dynamics predicted VWM precision on a trial-by-trial basis (Figure 2). To this end, pupil constriction during encoding and pupil dilation during prioritization were entered into an LME to predict the absolute hue error of the response at the end of the trial (also see Supplementary Materials). We found that both pupil constriction, *β* =.001 ± .0004, *t* = 2.45, *p* = .014, and pupil dilation amplitudes significantly predicted the error in hue at the end of the trial, *β* =.002 ± .0006, *t* =2.68, *p* =.007. Specifically, stronger pupil constriction during encoding and more pronounced pupil dilation elicited by internal prioritization predicted more precise responses (i.e. smaller absolute errors). Trial number did not significantly predict the precision of VWM reports, *β* =.005 ± .0028, *t* =1.66, *p* =.097. The model was determined using AIC-based backward selection, favoring a model that included neither baseline pupil size nor the interaction between encoding constrictions and prioritization dilations (Supplementary Materials). The fact that baseline pupil size and the interaction term did not meaningfully explain additional variance shows that 1) baseline pupil size was not significantly predictive of VWM precision when controlling for trial number and 2) external and internal attention may not interact to shape VWM precision. Together, these analyses indicate that pupil size indexes the intensity of external and internal attention, and that these components idiosyncratically predict the quality of VWM representations.

Errors significantly differed between the probe conditions, *t*s > 2.2, *p*s < .024 (bottom right in Figure 1). More specifically, whenever participants were probed with the exact color that was retro-cued, the absolute error was smaller compared with the other conditions. This is not surprising, since participants could refresh their representation of the color shortly before having to reproduce it. Note that the predictive pupil effects reported above are not driven by the probe conditions because probe types were considered in the analysis.

## 4. Discussion

Here, we found that the intensities of internal and external attention independently contribute to the quality of visual working memory content. Using openly available data (Wilschut & Mathôt, 2022), we show that the intensities of both types of attention are captured by distinct pupillary responses.

Recent studies employing subtle gaze biases (<1°) to track the focus of internal attention support the idea that internal attention is deployed at different intensities (de Vries & van Ede, 2023; Liu et al., 2023; Liu et al., 2022; van Ede et al., 2020; van Ede et al., 2019). The extent of these gaze biases toward internally attended content is associated with faster responses, indicating varying intensities of internal attention (van Ede et al., 2020; van Ede et al., 2019). Our study extends such findings by demonstrating that the intensity of internal attention, as measured through pupillary responses, enhances the precision of stored representations.

Turning to external attention, we report that smaller pupils during encoding lead to more precise VWM representations, complementing our previous work (Koevoet, Naber, et al., 2023). Our findings are consistent with the idea that distinct pupillometric response components inform about different operations of VWM such as encoding (external attention) and prioritization (internal attention) (Koevoet, Strauch, Van der Stigchel, et al., 2023; Strauch, Wang, et al., 2022). As such, relatively early pupil constrictions reveal encoding, while dilation responses to retro-cues reflect prioritization. Our approach overcomes inherent limitations of behavioral measures that cannot separate and quantify contributions of external and internal attention.

Using pupillary responses, we show that encoding and prioritization dynamically shape the quality of VWM representations. The two pupil response components explained idiosyncratic variation in VWM precision, and did not interact significantly. This shows that internal and external attention may idiosyncratically enhance stored material in a ‘more is better’ fashion – possibly without interacting strongly. These distinct pupillary response components serve as indices of the intensities of internal and external attention. Using (de)convolutional modeling techniques (see below), future work may provide more detailed insights into the interplay between external and internal attention on a trial-by-trial level.

Why is it beneficial to deploy attention at differing intensities? Deploying attention is often considered costly (Just et al., 2003; Kahneman, 1973; Koevoet, Strauch, Naber, et al., 2023), and stronger, more intense deployments of attention should be more expensive. To optimize such costs, the intensities of external and internal attention should be dynamically adjusted to only be deployed as strongly as necessary for a task. In contrast, an all-or-none deployment of attention does not allow for such a cost-efficient mechanism. The current data cannot test this costs-perspective directly as only naturally occurring fluctuations of internal and external intensities were investigated (e.g. due to fluctuations in the efficiency of attentional shifting across trials or failures to engage in the task, see De Jong et al., 1999). Thus, future work could experimentally manipulate task demands to elucidate whether the intensity of attention can be flexibly adjusted to optimize costs based on the current goals.

Two other potentially relevant pupillary dynamics were not directly assessed here: Baseline pupil size and pupil dilation during maintenance. First, baseline pupil size reveals tonic fluctuations in arousal, and has been linked to VWM precision/performance previously (Galeano-Keiner et al., 2023; Koevoet, Naber, et al., 2023; Robison & Unsworth, 2019; Starc et al., 2017). Specifically, baseline pupil size is thought to be linked to VWM precision following an inverted-U curve (Aston-Jones & Cohen, 2005; Gilzenrat et al., 2010; Jepma & Nieuwenhuis, 2011; Koevoet, Naber, et al., 2023; Koevoet, Strauch, Van der Stigchel, et al., 2023; Teigen, 1994; Yerkes & Dodson, 1908). Baseline pupil size did not affect VWM precision when controlling for trial number in the current data (Supplementary Materials). This could have multiple reasons: 1) Participants were at the performance optimum around the peak of the inverted-U curve. This is feasible since responses were overall very precise. 2) Trials were relatively long (7.7s) leading to reduced predictive power of baseline pupil size or 3) encoding constrictions and prioritization dilations are stronger predictors of VWM precision, accounting for similar variance as baseline pupil size.

Second, memorizing more items causes increased pupil dilation until one’s (V)WM capacity is reached (Beatty, 1982; Granholm et al., 1996; Kahneman & Beatty, 1966; Kosachenko et al., 2023; Robison & Unsworth, 2019; Zhou et al., 2022). Note that this dilatory pupil effect is also elicited in tasks that do not employ retro-cues (i.e. no internal prioritization is facilitated). This component is difficult to isolate in the current data because a caveat to utilizing pupillary dynamics to capture distinct processes is the temporal sluggishness of the signal. When (cognitive) processes occur in close temporal proximity to one another, delineating between the effects of pupil responses during encoding, maintenance and prioritization becomes complex (Mathôt & Vilotijević, 2022). To fully capture encoding, maintenance and prioritization in a single task on a trial-by-trial basis, trials need to be relatively slow-paced, allowing for the pupil to resolve cognitive events. The sluggishness of the pupil response could also be accounted for using (de)convolutional modeling techniques (Denison et al., 2020; Knapen et al., 2016; Korn & Bach, 2016; Wierda et al., 2012). Future work could incorporate such methods and/or techniques to obtain a complete picture of all cognitive operations employed when flexibly using VWM.

Together, our findings show that the intensities of internal and external attention are captured by pupil size on a trial-by-trial basis. The intensity of internal and external attention independently predict the quality of VWM representations. Our approach holds promise to elucidate the intricate interplay between internal and external attention to effectively guide behavior in the rich visual world.

## Data Accessibility Statements

Analyses scripts to reproduce the results are available via the Open Science Framework: https://osf.io/ek3ap/. The original analyses and data by Wilschut and Mathôt (2022) can be found here: https://osf.io/qksfh/.

## Additional File

The additional file for this article can be found as follows:

10.5334/joc.336.s1 Supplementary Material. The Intensity of Internal and External Attention Assessed with Pupillometry.
