# Supplementary material for: The Intensity of Internal and External Attention Assessed with Pupillometry
Source: J Cogn. 2024 Jan 9;7(1):8. doi: 10.5334/joc.336 (PMC10786008; doi:10.5334/joc.336)
Supplement: Supplementary Material. — The Intensity of Internal and External Attention Assessed with Pupillometry. [file joc-7-1-336-s1.pdf]

Supplement to: *The Intensity of Internal and External Attention Assessed with Pupillometry*

Damian Koevoet*, Marnix Naber , Christoph Strauch , and Stefan Van der Stigchel

Experimental Psychology, Helmholtz Institute, Utrecht University, The Netherlands

*Correspondence to d.koevoet@uu.nl

1 Model Selection & Outcomes

Models were compared based on the Akaike information criterion. These different models were theoretically and methodologically meaningful, and always included the two main predictors of interest: encoding constriction and prioritization dilation. Probe type was added as a covariate in all models to ensure that this factor would not drive potential predictive effects of the pupillary responses. Similarly, we added trial number as a covariate in order to control for the fact that baseline pupil size decreases over time (Strauch et al., 2022).

We followed recommendations from Barr (2013) when fitting the linear mixed-effects models. It is normally recommended to fit random intercepts per participant as well as random slopes for predictor variables to limit possible Type 1 errors. However, convergence is a prerequisite to reliably interpret estimates from linear mixed-effects models (Barr, 2013). Since the model(s) that modeled random slopes did not convergence, we opted to ommit the random slopes term for probe types in the model comparisons below.

Below are the models that were compared based on complexity and fit:

- m1: absolute hue error ~ encoding constriction * prioritization dilation * baseline pupil size * trial number + probe type + (1|participant)
- m2: absolute hue error ~ encoding constriction * trial number + prioritization dilation * trial number + baseline pupil size * trial number + probe type + (1|participant)
- m3: absolute hue error ~ encoding constriction * trial number + prioritization dilation * trial number + baseline pupil size + probe type + (1|participant)
- m4: absolute hue error ~ encoding constriction + prioritization dilation * trial number + baseline pupil size + probe type + (1|participant)
- m5: absolute hue error ~ encoding constriction * prioritization dilation * baseline pupil size + trial number + probe type + (1|participant)
- m6: absolute hue error ~ encoding constriction * prioritization dilation + baseline pupil size + trial number + probe type + (1|participant)
- m7: absolute hue error ~ encoding constriction + prioritization dilation + baseline pupil size + trial number + probe type + (1|participant)
- m8: absolute hue error ~ encoding constriction + prioritization dilation + trial number + probe type + (1|participant)
- m9: absolute hue error ~ encoding constriction + prioritization dilation + probe type + (1|participant)

We selected 'm8' based on the lowest AIC value compared to the other models (Table 1). Note that if one would select based on Log-Likelihood, 'm9' would be chosen which shows the same effects conceptually.

For completeness, the full results of the chosen model are provided below in Table 2.

Table 1: Model fit comparisons.

Model	AIC	Log-likelihood	nPar
m1	45658.36	-22807.18	22
m2	45645.10	-22808.55	14
m3	45643.77	-22808.89	13
m4	45641.78	-22808.89	12
m5	45646.11	-22808.05	15
m6	45642.30	-22809.15	12
m7	45640.73	-22809.36	11
**m8**	**45638.73**	**-22809.36**	**10**
m9	45639.48	-22810.74	9

Note. nPar indicates the number of parameters included in the model.

Table 2: Full outcomes of m8.

**Predictor**	**Beta**	**SE**	***t***	***p***
Intercept	11.57	.658	17.58	<.001
Probe type (Exact vs. Between)	1.82	.479	3.81	<.001
Probe type (Exact vs. Different)	1.09	.478	2.27	.023
Probe type (Exact vs. None)	1.68	.478	3.51	<.001
Probe type (Exact vs. Within)	2.00	.479	4.18	<.001
Encoding constriction	.001	.0004	2.45	.014
Prioritization dilation	-.002	.0006	2.68	.007
Trial number	-.005	.003	1.66	.097
Participant	2.66	.074		

References

Barr, D. (2013). Random effects structure for testing interactions in linear mixed-effects models. *Frontiers in Psychology*, *4*. https://doi.org/10.3389/fpsyg.2013.00328

Strauch, C., Wang, C.-A., Einhäuser, W., Van der Stigchel, S., & Naber, M. (2022). Pupillometry as an integrated readout of distinct attentional networks. *Trends in Neurosciences*, *45*(8), 635–647. https://doi.org/10.1016/j.tins.2022.05.003
